# Biomarkers for Severity of Spinal Cord Injury in the Cerebrospinal Fluid of Rats

**DOI:** 10.1371/journal.pone.0019247

**Published:** 2011-04-29

**Authors:** Joanna M. Lubieniecka, Femke Streijger, Jae H. T. Lee, Nikolay Stoynov, Jie Liu, Randy Mottus, Tom Pfeifer, Brian K. Kwon, Jens R. Coorssen, Leonard J. Foster, Thomas A. Grigliatti, Wolfram Tetzlaff

**Affiliations:** 1 International Collaboration on Repair Discoveries (ICORD), Blusson Spinal Cord Centre, University of British Columbia, Vancouver, British Columbia, Canada; 2 Centre for High-Throughput Biology and Department of Biochemistry and Molecular Biology, University of British Columbia, Vancouver, British Columbia, Canada; 3 Department of Zoology, Life Sciences Institute, University of British Columbia, Vancouver, British Columbia, Canada; 4 Centre for Drug Research and Development (CDRD), University of British Columbia, Vancouver, British Columbia, Canada; 5 Molecular Physiology Department, School of Medicine, University of Western Sydney, Penrith, New South Wales, Australia; 6 Department of Orthopaedics, University of British Columbia, Vancouver, British Columbia, Canada; Virginia Commonwealth University Rehabilitation and Research Center, United States of America

## Abstract

One of the major challenges in management of spinal cord injury (SCI) is that the assessment of injury severity is often imprecise. Identification of reliable, easily quantifiable biomarkers that delineate the severity of the initial injury and that have prognostic value for the degree of functional recovery would significantly aid the clinician in the choice of potential treatments. To find such biomarkers we performed quantitative liquid chromatography-mass spectrometry (LC-MS/MS) analyses of cerebrospinal fluid (CSF) collected from rats 24 h after either a moderate or severe SCI. We identified a panel of 42 putative biomarkers of SCI, 10 of which represent potential biomarkers of SCI severity. Three of the candidate biomarkers, Ywhaz, Itih4, and Gpx3 were also validated by Western blot in a biological replicate of the injury. The putative biomarkers identified in this study may potentially be a valuable tool in the assessment of the extent of spinal cord damage.

## Introduction

It is estimated that over 30,000 people in Canada, and approximately ten times that many in the USA, are living with spinal cord injury (SCI), and more than 1,000 and 11,000 new cases occur annually in Canada and the USA, respectively (http://rickhansenregistry.org; https://www.nscisc.uab.edu).

Improvements in the medical, surgical, and rehabilitative care have dramatically extended the lifespan and increased the quality of life of individuals with acute and chronic SCI, but much remains to be improved in restoring function for individuals with SCI. Little can be done about the primary mechanical damage to the spinal cord that occurs on impact. However, most spinal cord injuries are anatomically incomplete and the secondary parenchymal damage contributes significantly to the final extent of neural damage and ultimately to the extent of the long term disability [1], [2], [3]. Due to its prolonged nature, this secondary injury is amenable to treatment, and a number of candidate neuroprotective interventions that may potentially halt or attenuate the secondary damage have been developed [4]. The severity of the initial injury likely plays a significant role in determining the nature and amplitude of the secondary response and thus the appropriate interventions. However, the current clinical measures for characterizing injury severity are based on functional tests that cannot be relied upon immediately following injury because they are often compromised by shock, other attendant injuries and drugs or alcohol.

Biomarkers are objectively quantifiable biological characteristics and represent a very attractive and unbiased tool for assessing SCI severity. Furthermore, markers associated with the pathophysiology of acute SCI should aid in monitoring the biological effects of a candidate treatment and also may identify potential targets for novel treatments that reduce secondary damage [5]. To date, few studies have been conducted to identify biomarkers of SCI, and most have investigated patients at risk of ischemic SCI after surgery for thoracic and thoracoabdominal aortic aneurysms (TAAAs), or after a nerve root compression in animal models [6]. Three studies have investigated biomarkers in traumatic animal SCI models, and two examined cerebrospinal fluid (CSF) from patients with traumatic SCI [7], [8], [9], [10], [11]. Collectively these studies have identified S100 calcium binding protein beta (S100ß), neuron specific enolase (NSE), neurofilament protein (NFL), myelin basic protein (MBP), glial fibrillary acidic protein (GFAP), the microtubule- associated tau proteins, monocyte chemotactic protein 1 (MCP-1), and interleukins 6 and 8 (IL-6 and IL-8) as potential markers of spinal cord damage [6], [11]. These studies need to be extended, validated and developed for their potential use in objectively determining the severity of SCI.

The goal of this study was to identify candidate biomarkers in the CSF that could be evaluated for their utility in characterizing the severity of damage that occurs due to the primary mechanical trauma. We used a preclinical rat model in which the spinal cord injury is induced via a precisely controlled electro-mechanic impactor. Due to its proximity to the CNS tissues we chose to look for candidate SCI biomarkers in CSF, rather than in blood. The application of liquid chromatography-mass spectrometry (LC-MS/MS) combined with stable isotope labeling enabled us to quantify changes in a large number of CSF proteins, yielding a panel of 42 candidate biomarkers of SCI, including 10 biomarkers that have the potential to distinguish between a moderate and severe injury.

## Materials and Methods

### Ethics Statement

All animal procedures were performed in accordance with the guidelines of the Canadian Council for Animal Care and approved by the University of British Columbia animal care committee (protocol # A09-0086).

### Animals

In total, 108 male Sprague–Dawley rats (Charles River Breeding Laboratories) 11–12 weeks old and weighing 350–420 g were used for this study. All animals were maintained in a standard housing environment with *ad libitum* access to food and water. Twenty seven animals were used for behavioral and histological assessment of the injury. A further 36 animals were used for CSF sample collection to search for biomarkers by LC-MS/MS. In an additional cohort of 45 animals, CSF samples were collected for biomarker validation by Western blot (WB). In all experiments the animals were randomized and equally divided into three experimental groups: (1) sham control, (2) moderate injury (1.3 mm displacement SCI) and (3) severe (1.7 mm displacement SCI).

### Surgery

Animals were anesthetized with an intraperitoneal (i.p.) injection of ketamine (70 mg/kg) and rompun (10 mg/kg). After inducing a surgical plane of anesthesia (end point of anesthesia was assessed by toe pinch and eye blink reflex), the surgical area was shaved and disinfected. The animal was placed in a stereotaxic apparatus (Kopf Instruments, Tujunga, CA) and a dorsal midline incision was made from C2 to C7. After the cervical vertebrae were carefully exposed laminectomy was performed with fine rongeur at the C4/5 level to expose the spinal cord. Following laminectomy, the spine was stabilized by clamping the transverse processes of one segment above and below the lesion site [12]. The contusion was generated using the displacement-controlled OSU impactor (Ohio State University, Columbus, Ohio) [13]. The impactor tip was centered over the left side of the C4/5 and aligned at the grey/white matter border. The contusion was done with displacement of 1.3 mm or 1.7 mm in the moderate and severe injury group, respectively. For sham treatment, rats received a laminectomy followed by clamping of the C3 and C5 transverse process and loading onto the impactor device, but did not receive a SCI.

After surgery, the animals were kept in a temperature-controlled incubator set at 32°C until they were completely awake. Subcutaneous administration of 10 ml of Ringer's-lactate solution and buprenorphine (0.03 mg/kg) was given to prevent dehydration and post-operative pain.

### Injury assessment

Initially 27 animals (sham: n = 4, 1.3-mm: n = 9, 1.7-mm: n = 14) were used for assessment of SCI severity. Behavioural assessment of forelimb function was conducted 1 week prior to injury and 1, 4, and 6 weeks after injury. To assess the overall level of functional impairment, rats were examined for forelimb use during vertical exploration in a cylinder [14], [15], [16]. On testing days, rats were individually placed and videotaped in a clear Plexiglas cylinder (20 cm in diameter and 30 cm high) for 2×5 minutes per session. Afterwards, the tapes were analyzed frame-by-frame by an observer blinded to the randomized treatment assignment of the animalsto determine forelimb usage for contacting the wall of the cylinder during 20 independent rears. During a rearing motion, the first forelimb to contact the wall was scored as an either “left”, “right”, or “both” movement. If an independent movement is followed by placement of the other limb on the wall without first removing the first paw, this would be scored as a subsequent “both” movement. After scoring a “both” movement, every additional combination of two-limb movements received a subsequent “both” score. Forelimb usage was expressed as percent use of the impaired paw (sum of left and both) relative to the total wall placements.

At the end of the experiment (6–10 wks post-injury), animals were perfused intra-cardially with 1×PBS followed by 4% paraformaldehyde. Cervical spinal cord were collected and post-fixed overnight in 4% paraformaldehyde. After cryoprotection in 12%, 18% and 24% sucrose, spinal cords were frozen and cut in 20 µm thick cross-section on a cryostat (Microm, Heidelberg, Germany). To visualize white and grey matter, coronal sections through the cervical injury site were stained with Eriochrome–cyanine (EC) as described previously [17]. Briefly, frozen spinal cord sections were rehydrated and stained in EC Solution (0.16% EC, 0.4% sulfuric acid and 0.4% ferric chloride) at room temperature for 10 min. After a gentle rinse in dH_2_O, slides were differentiated in 0.5% ferric ammonium sulfate at room temperature for ∼2 min., counterstained with 1% Neutral Red and cover-slipped using Entallan mounting medium (EM Science, Gibbstown, NJ). Images were captured with a bright-field microscope (5× objective, Leica DM5000 B microscope, Leica Microsystems). The section with the least amount of white matter sparing was defined as the lesion epicenter.

For the cohort of animals used in biomarker discovery and validation the actual force delivered was recorded for each impact which, in combination with a visual inspection (that shows a hematoma), allowed the surgeon to confirm the impact. Criteria for the force curves were set and any spikes (bone hits) or unusual force clips (indicative of slippage) would lead to exclusion of the animal from the study. Recorded force of impact was also used to assess injury severity and animals that received an impact with recorded force outside the range of 150–161 Kdynes for moderate injury or 165–176 Kdynes for severe injury were excluded from the study. Using these criteria seven animals were removed from the biomarker study after injury because of either insufficient or excessive severity. Two animals did not wake up from anesthesia. Thus, 32 and 40 animals were used for sample collection for LC-MS/MS and Western blot analyses, respectively.

### Cerebrospinal fluid (CSF) collection

CSF was collected by puncture of the cisterna magna 24 h after injury. To minimize protein degradation in tissues, anesthetized rats were perfused intracardially with 100 ml of ice cold PBS prior to CSF collection and were kept on ice during sample collection. CSF was visually inspected for the presence of blood contamination and contaminated samples were excluded from further analysis (5 CSF samples from LC-MS/MS analysis and 4 samples from WB analysis) leaving samples from 27 animals for LC-MS/MS analysis and 36 animals for WB analysis. CSF samples were centrifuged at 6,000×g at 4°C to remove cellular debris. Protease inhibitors (“Complete” Protease Inhibitor Cocktail, Roche Diagnostics Canada, Laval, Quebec) were added to the pre-cleared CSF and the samples were frozen at −80°C.

### Protein preparation for mass spectrometry (MS)

**Figure 1:**
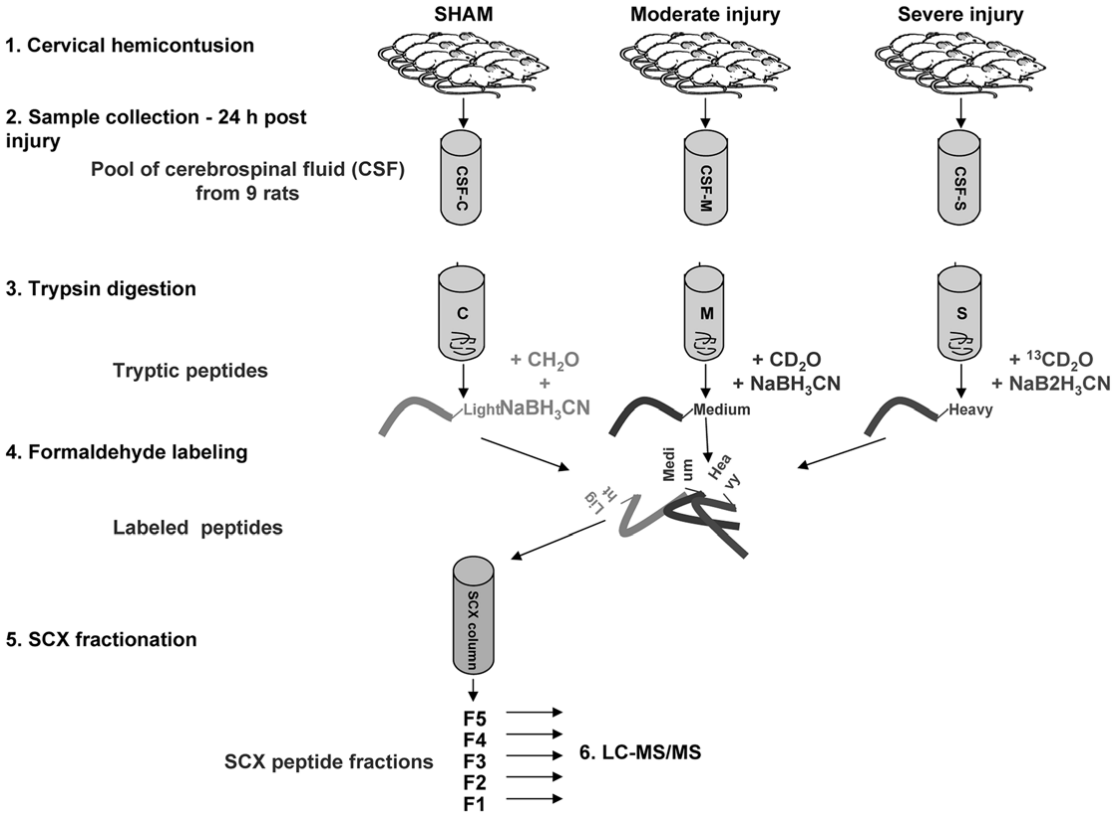
Experimental Outline.

For MS analysis, CSF was thawed on ice and all samples within each treatment group were combined to give a single pooled CSF sample for each treatment (Figure 1). The protein concentration of each pooled CSF sample was determined using EZQ Protein Quantitation kit (Molecular Probes, Eugene, OR) and aliquots containing 25 µg of protein were precipitated using the ethanol/acetate method [18]. The precipitated protein pellet was air-dried and re-dissolved in 30 µl of trypsin digestion buffer (1% sodium deoxycholate, 50 mM NH_4_HCO_3_) for MS analysis.

The protein samples were denatured for 5 min at 99°C, reduced with 0.5 µg Dithiothreitol (DTT) for 30 min at 37°C, alkylated with 2.5 µg iodoacetamide (20 min at 37°C), and digested with 0.5 µg trypsin (overnight at 37°C). Following digestion, peptides were desalted using C_18_STop And Go Extraction (STAGE) tips (made “in-house”) [19]. Next, the peptides from CSF of animals after sham, moderate, and severe injury were labeled with light (CH_2_O), medium (CD_2_O), and heavy (^13^CD_2_O) isotopologues of formaldehyde, respectively [20]. The use of different labels enabled mixing of peptides from CSF of animals after sham, moderate and severe injury and simultaneous sample processing and analysis, thus eliminating artificial differences due to between run variability. The labeled peptides were then fractionated on C_18_-SCX-C_18_ STAGE tips using a 5-step ammonium acetate elution gradient [21] and the dried peptide fractions were resuspended in 1% trifluoroacetic acid, 3% acetonitrile, and 0.5% acetic acid.

### Liquid chromatography-mass spectrometry (LC-MS/MS)

The peptide fractions were analyzed on a linear trapping quadrupole-OrbitrapXL hybrid mass spectrometer (ThermoFisher Scientific, Waltham, MA, USA) as previously described [22]. The LTQ-OrbitrapXL was on-line coupled to an 1100 Series nanoflow HPLC (Agilent Technologies, Santa Clara, CA) using a nanospray ionization source (ProxeonBiosystems, Odense, Denmark) holding columns packed into 15-cm-long, 75-µm-inner diameter fused silica emitters (8-µm-diameter opening, pulled on a P-2000 laser puller from Sutter Instruments) using 3-µm-diameter ReproSilPur C18 beads. Buffer A consisted of 0.5% acetic acid, and buffer B consisted of 0.5% acetic acid and 80% acetonitrile. Gradients were run from 6% B to 30% B over 90 min, then 30% B to 80% B in the next 10 min, held at 80% B for 5 min, and then dropped to 6% B for another 15 min to recondition the column. The mass spectrometer was set to acquire a full-range scan at 60,000 resolution from 350 to 1500 Thomson (Th) units in the Orbitrap and to simultaneously fragment the top five peptide ions in each cycle in the LTQ. Fragment spectra of multiply charged ions were then searched against the Rat International Protein Index database with reversed sequences concatenated onto it (v3.47, 80,285 sequences) using MASCOT v2.2 (Matrix Science, London). The following search parameters were used in all MASCOT searches: trypsin (allowing up to one missed cleavage) or no enzyme specificity (in separate searches); carbamidomethyl as a fixed modification, variable modifications of dimethylation by both hydrogen isotopes at the peptides' amino termini and lysine ε-amino groups, 10 ppm peptide tolerance; 0.8 daltons (Da) fragment ion tolerance, and electrospray ionization-Trap fragmentation characteristics. All identified peptides were first recalibrated using MSQuant [23] and then only proteins with at least two unique peptide sequences, IonsScores greater than 30 and an absolute calibrated mass error of <5 ppm were considered identified. The false discovery rate for this dataset, estimated at <1%, was calculated by dividing the sequence-reversed proteins that failed to be eliminated after applying the above criteria. Results from multiple experiments were combined at the peptide level and then recombined into a final, non-redundant list of identified proteins. Chromatographic peak areas for the formaldehyde isotopologues (light, medium and heavy) of each detected peptide were first extracted using MSQuant [23] and then manually inspected to correct errors from overlapping peaks, missed monoisotopic peaks, etc. Only proteins for which a quantitative ratio was detected in at least two experiments were considered further. Peptides matching to multiple known isoforms/splice variants were retained and used for quantifying in each instance.

### Western Blotting

CSF samples from four rats in each treatment group were pooled randomly to obtain sufficient protein for analysis. This sample pooling created three CSF samples for each treatment (C1, C2, C3 – sham; M1, M2, M3 – moderate injury; S1, S2, S3 – severe injury). Twenty micrograms of total protein was resuspended in 1× sodium dodecyl sulfate (SDS) buffer (62.5 mMTris–HCl, pH 6.8, 10% glycerol, 2% SDS, 5% 0.1 M DTT, 0.05% bromophenol blue), loaded on a 12% or 7% SDS-polyacrylamide gel and resolved by electrophoresis at 90 V (4°C). Proteins were electrotransfered to a 0.5 µm nitrocellulose membranes at a constant current of 180 mA for 60 min in 4°C. For S100a8 immunobloting, proteins were separated on 18% gel and the WB was done essentially as described in Coorssen et al [24], except that myoglobin was not added to the protein sample or the gel equilibrating buffer. Membrane washing and protein detection were done according to manufacturer's recommendations for the Odyssey and IRDye system (LI-COR Biosciences, Lincoln, Nebraska), and the following antibodies were used at 1∶200 dilution: Ywhaz (Abcam, #ab32622), Gpx3 (Abcam, #ab59524), S100a8 (Santa Cruz, #sc-8113), Itih4 (Santa Cruz, #sc-34471), and Trf (Santa Cruz, #sc-22597).

### Ingenuity Pathways Analysis

The functional analyses were generated through the use of Ingenuity Pathways Analysis (IPA) v7.6 (www.ingenuity.com). For this analysis the average relative protein ratios obtained from MS analysis are converted to fold change values by the IPA software. The data set was filtered for proteins present in CSF only, and proteins from the filtered dataset that met the fold change cut-off of 1.5, and were associated with biological functions in the Ingenuity Pathways Knowledge Base, were considered for the analysis. The IPA uses right-tailed Fischer's exact test to calculate a p-value determining the probability that each biological function assigned to that data set is due to random chance alone. The p-value for a given function is calculated by considering the number of proteins in the dataset that participate in that function and the total number of molecules that are known to be associated with that function in the IPA knowledge base. P-values<0.05 were considered statistically significant, non-random associations.

## Results

**Figure 2:**
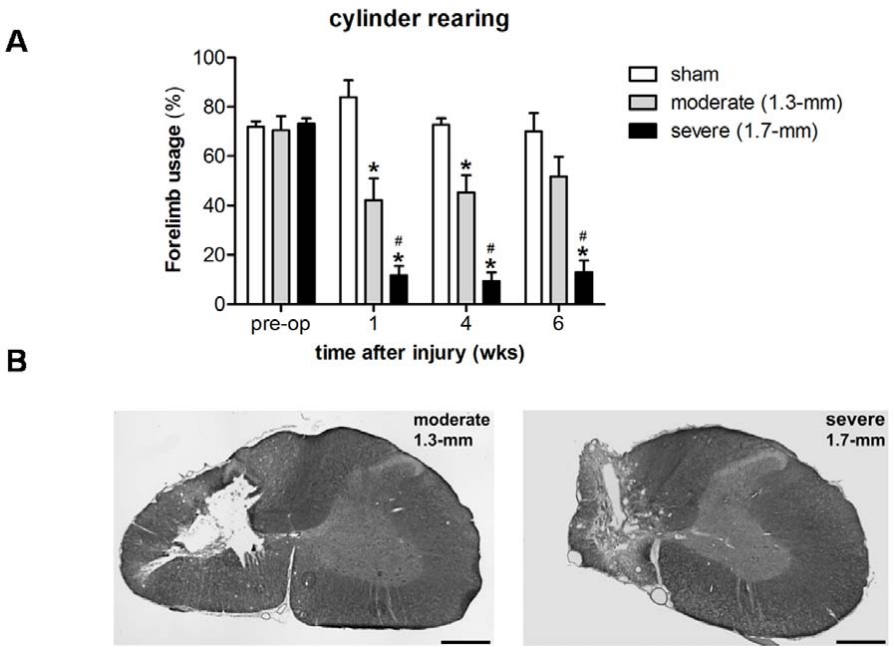
Injury assessment. (A) Influence of 1.3-mm and 1.7-mm displacement injuries at C5 on forelimb usage during rearing. A contusion injury with a tissue displacement of 1.3-mm produced a ‘mild’ injury with forelimb motor deficits that recovered over time. The more ‘severe’ 1.7-mm displacement injury produced nearly complete loss of forelimb usage that was sustained until the end of the study. Error bars indicate standard error of the mean (SEM). “*” significantly different from sham group, p≤0.05; # 1.7-mm groups significantly different from 1.3-mm group, p≤0.05. (B) Histological outcome to the spinal cord following contusion injury with 1.3-mm or 1.7-mm tissue displacement 6–10 weeks after injury. Example image of eriochrome cyanine- and neutral red-stained coronal section of the injury at the epicenter. Scale bar: 500 µm.

Our objective was to create a unilateral cervical contusion injury with defined tissue displacements (1.3 and 1.7 mm) to produce injuries of graded severity (“moderate and “severe”). Rats receiving contusion injuries targeted at C4/C5 with displacement set to 1.3-mm yielded an average force recording of 155.7±5.0 Kdynes. An average force of 170.6±5.0 Kdynes was reached with a displacement setting of 1.7 mm. Preoperatively, non-contused (sham) animals and rats from the moderate (1.3-mm) and severe (1.7-mm) injury groups performed equally well (Figure 2a) and used their impaired forelimb either independently or in combination with the contralateral paw ∼70% of total wall placements made (forelimb usage sham: 71.9±6.1%, moderate: 70.6±5.6%, severe: 73.2±2.1%). Postoperatively, animals from both the moderate and severe injury group were significantly different compared to non-contused sham controls during the 6 week time period they were observed (ANOVA repeated measures with Bonferroni correction, moderate: p = 0.032, severe: p = 0.0001, Figure 2a). Group comparison per week (One-way ANOVA) revealed that the moderate injury produced forelimb deficits at weeks 1 and 4 after injury (wk1: 42.2±8.8%; wk4: 45.4±6.9%) compared to sham animals (wk1: 83.9±6.9%, p = 0.001; wk4: 72.8±2.6%, p = 0.02). However, the animals did recover over time resulting in an average forelimb usage of 51.8±8.0% at 6 week following injury (sham: 70.1±7.5, p = 0.0413). The severe injury group was significantly different from both sham and moderate injury animals at any time point following injury and only used their impaired forelimb during ∼10% of the total placements made (wk1: 11.7±3.8%; wk4: 9.4±3.4%; wk6: 13.0±4.7%).

Coronal sections of the spinal cord taken from animals receiving either moderate or severe displacement injuries are shown in Figure 2b. At the epicentre, the moderate displacement injury mostly encompassed the grey matter, with the exception of a small rim of spared tissue of the dorsal horn. Only minimal ipsilateral white matter damage was detected to the ventrolateral, lateral, and dorsolateral funiculus. The severe injury group showed severe damage of ipsilateral white matter compared to the moderate injury group and with only a small rim of spared white matter of the ventrolateral and dorsal funiculus. The severe displacement injury completely destroyed the dorsal and ventral horns at the epicentre.

In order to quantify changes in protein composition in the rat CSF 24 hours after SCI, we applied quantitative proteomics using stable isotope labeling and LC-MS/MS. This proteomic approach allowed for simultaneous analysis of CSF from sham, moderate, and severe injury groups. Prior to LC-MS/MS analyses the CSF samples were fractionated by strong cation exchange (SCX) to decrease the complexity of CSF samples. Due to the low volume of CSF obtained from a single animal (50–80 µl) and the relatively low protein concentration in the CSF (0.2–0.8 µg/µl), the samples in each treatment group were pooled to attain sufficient protein for subsequent fractionation and MS analysis.

**Figure 3:**
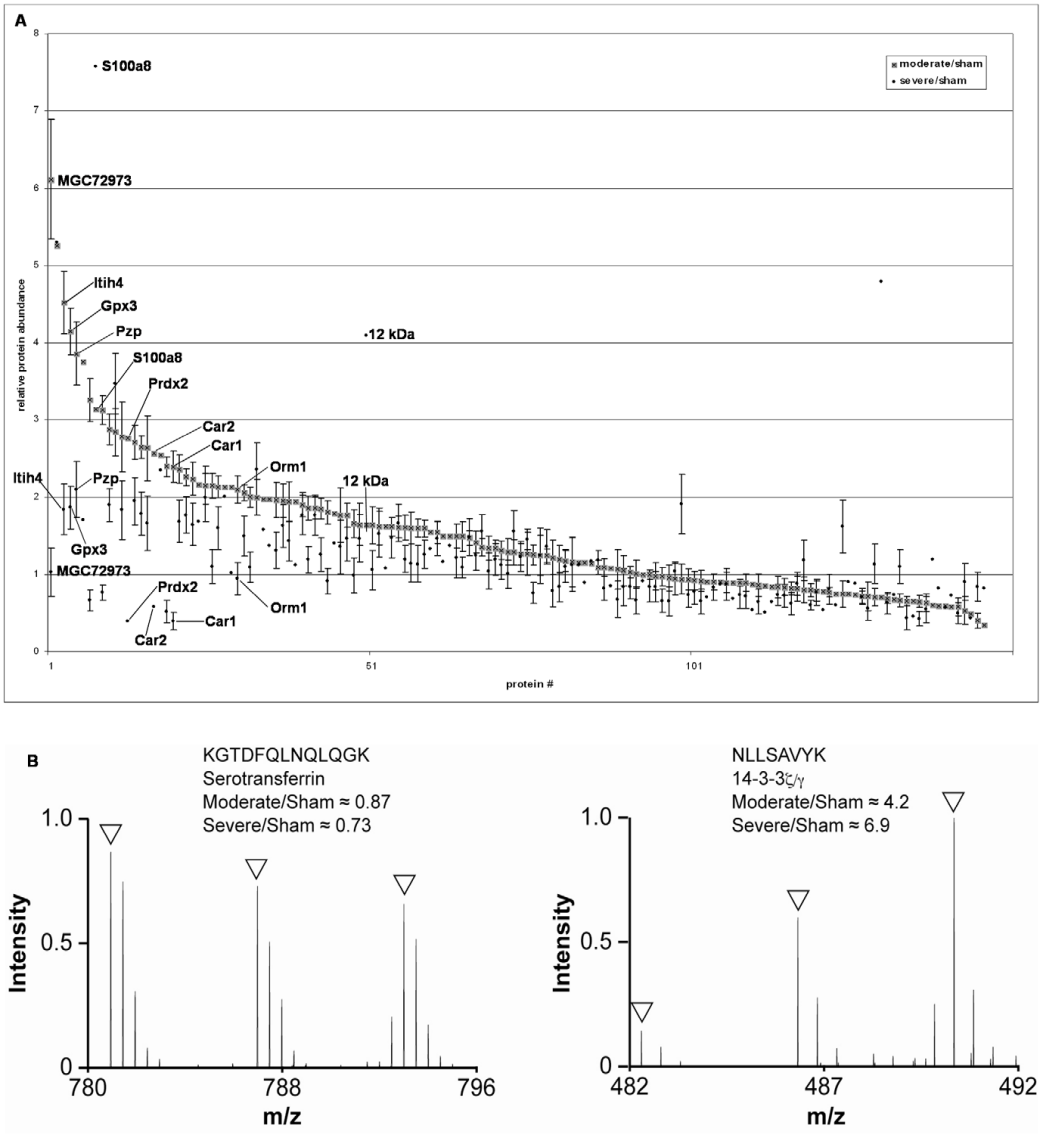
Quantitative proteomic analysis of CSF from injured animals. (a) Relative protein abundances for all proteins detected in rat CSF 24 h after sham, moderate or severe SCI. Labeled data points represent potential biomarkers of SCI severity (Tables 3 and 4). Error bars represent standard error of the mean (SEM). (b) Representative examples of MS spectra from the indicated peptides. Open arrowheads point to the monoisotopic peaks from light (sham), medium (moderate) and heavy (severe) conditions.

Each pooled sample was split into 3 aliquots and analyzed by LC-MS/MS on separate days. The LC-MS/MS identified 2,924 peptides (Table S1), which after applying the cut-off criteria (see Materials and Methods), resulted in identification of 334 proteins in the rat CSF (Table S2). Of these 334 proteins, 146 were consistently quantifiable across at least two MS runs and are presented as moderate injury/sham and severe injury/sham ratios (Table S3, Figure 3).

Potential biomarkers of SCI were defined using a cut-off ratio of more than 2.0 or less than 0.5. Using this cut off, 42 potential biomarkers of SCI were identified (Tables 1,2, 3, 4). Proteins that showed at least a 2-fold difference in ratio between the moderate and severe injury models were defined as putative biomarkers of SCI severity. Using these selection criteria, 10 of the 42 candidate biomarkers appeared to distinguish between moderate and severe SCI (Tables 3 and 4, Figure 3).

**Table 1:** Potential biomarkers of SCI identified by quantitative LC-MS/MS analysis of CSF from rats following experimental SCI.

	Protein ID	Protein Description	Protein Symbol	Moderate/sham		Severe/sham		Gene Ontology
				AVG ratios	SEM	AVG ratios	SEM	
1	IPI00781399	Immunoglobulin heavy chain 1a	LOC678701	**2.9**	0.20	**1.9**	0.22	rat plasma protein; immune response
2	IPI00324893	14-3-3 protein zeta/delta	Ywhaz	**2.8**	0.31	**3.5**	0.40	histamine secretion; protein targeting to mitochondrion; response to drug
3	IPI00568389	similar to NGF-binding Ig light chain	LOC500183	**2.8**	0.45	**1.8**	0.38	Immune response
4	IPI00188338	inter-alpha-trypsin inhibitor 1	Itih1	**2.7**	0.22	**2.0**	0.31	hyaluronan metabolism, leukocyte activation
5	IPI00197703	Apolipoprotein A-I	Apoa1	**2.6**	0.15	**1.8**	0.28	signal transduction; adrenal gland development; axon regeneration; endothelial cell migration and proliferation; cholesterol transport and metabolism; glucocorticoid metabolic process; lipid storage and metabolism; lipoprotein metabolic processes; negative regulation of cytokine secretion; regulation of hydrolase activity; regulation of lipoprotein remodeling; organ regeneration; phosphatidylcholine biosynthetic process; phospholipid transport and metabolism; regulation of transferase activity; protein stabilization; regulation of amino acid phosphorylation; response to drug; steroid metabolic process; response to estrogen stimulu
6	IPI00372372	Serine (Or cysteine) peptidase inhibitor C1 (Antithrombin)	Serpinc1	**2.6**	0.42	**1.7**	0.35	negative regulation of inflammatory response; response to nutrient
7	IPI00210900	alpha-1-microglobulin/bikunin precursor	Ambp	**2.4**	0.18	**1.7**	0.28	protein-chromophore linkage; transport; cell adhesion; pregnancy; heme catabolic process; interspecies interaction; negative regulation of JNK cascade; negative regulation of immune response
8	IPI00389806	similar to inter-alpha-inhibitor H2 chain	LOC498793	**2.3**	0.11	**1.8**	0.24	serine protease inhibitor activity; hyaluronan metabolism
9	IPI00565114	Junctional adhesion molecule 2 (13 kDa protein)	Jam2	**2.2**	0.21	**1.6**	0.28	Cell-cell adhesion; (may be involved in maintenance of stem cell identity)
10	IPI00559019	similar to Igκ	13 kDa protein	**2.2**	0.25	**2.0**	0.31	immune response
11	IPI00188225	C-reactive protein	Crp	**2.1**	0.14	**1.6**	0.28	acute-phase response; complement activation (classical pathway); negative regulation of foam cell differentiation; negative regulation of lipid storage; transport
12	IPI00231783	L-lactate dehydrogenase B chain	Ldhb	**2.0**	0.24	**2.4**	0.35	NAD metabolic process; anaerobic glycolysis; cellular carbohydrate metabolic process; lactate metabolic process; oxidation reduction
13	IPI00778633	Apolipoprotein H	Apoh	**2.0**	0.22	**1.6**	0.28	blood coagulation; negative regulation of angiogenesis; negative regulation of endothelial cell migration and proliferation; negative regulation of fibrinolysis; negative regulation of myeloid and smooth muscle cell apoptosis; organ regeneration; plasminogen activation; positive regulation of lipoprotein lipase activity; triglyceride transport and metabolic process
14	IPI00191139	Secretogranin-3	Scg3	**0.7**	0.06	**0.4**	0.15	not annotated
15	IPI00388323	heparan sulfate proteoglycan 2	Hspg2	**0.6**	0.08	**0.4**	0.09	embryo implantation; negative regulation of cell adhesion; regulation of cell proliferation; organ regeneration; response to drug; response to hypoxia
16	IPI00331757	FAM3C-like protein	Fam3c	**0.6**	0.12	**0.5**	0.17	not annotated
17	IPI00197041	Neuroblastoma suppressor of tumorigenicity 1	Nbl1	**0.6**	0.11	**0.5**	0.14	down-regulated in transformed cells (possible tumor suppressor)
18	IPI00392886	Alpha-2-macroglobulin	A2m	**2.1**	0.16	**1.1**	0.22	acute-phase response; inflammatory response; negative regulation of complement activation (lectin pathway); response to chemical stimulus; response to glucocorticoid stimulus; response to nutrient; response to wounding
19	IPI00679245	T-kininogen 2	MGC108747	**2.0**	0.13	**1.1**	0.20	acute-phase response; vasodilation
20	IPI00480639	Complement C3	C3	**2.0**	0.23	**1.3**	0.23	blood coagulation; complement activation; inflammatory response; positive regulation of phagocytosis; positive regulation of type IIa hypersensitivity
21	IPI00204359	Beta-2-microglobulin	B2m	**0.5**	0.18	**0.9**	0.24	antigen processing and presentation; positive regulation of T cell mediated cytotoxicity; response to cadmium ion; response to drug

Proteins consistently changed across three LC-MS/MS runs.

Potential biomarkers were selected based on average ratio ≥2.0 or ≤0.5. AVG – average; SEM – standard error of the mean.

**Table 2:** Potential biomarkers of SCI identified by quantitative LC-MS/MS analysis of CSF from rats following experimental SCI.

	Protein ID	Protein Description	Protein symbol	Run 1 ratios (±SD)		Run 2 ratios (±SD)		Average ratios		Gene Ontology
				M/S	S/S	M/S	S/S	M/S	S/S	
1	IPI00230835	14-3-3 protein gamma	Ywhag	**4.46±0**	**7.1±0**	**6.06±0**	**3.5±0**	**5.3**	**5.3**	protein targeting; neuron differentiation; synaptic plasticity; signal transduction
2	IPI00515829	Kininogen 1	Kng1	**3.33±0**	**2.05±0**	**3.27±0**	**2.99±0**	**3.3**	**2.5**	elevation of cytosolic Ca^2+^; MAP kinase activity; coagulation; cell adhesion; lymphocyte proliferation; proteolysis; MAPKKK cascade; endothelial cell proliferation; fibroblast proliferation; endopeptidase activity
3	IPI00204451	Complement factor I	Cfi	**1.45±0.19**	**1.77±0.46**	**2.81±1.24**	**2.25±0.46**	**2.1**	**2.0**	complement activation; innate immune response; proteolysis
4	IPI00391528	Similar to immunoglobulin Kappa light chain V gene	13 kDa protein	**2.36±0**	**1.82±0**	**1.66±0**	**1.09**	**2.0**	**1.5**	immune response
5	IPI00189981	Prothrombin (Fragment)	F2	**1.98±1.15**	**2.59±0**	**1.97±0**	**0.57±0.2**	**2.0**	**1.6**	acute phase response; coagulation; cell surface receptor linked signal transduction; cytosolic Ca^2+^ homeostasis; fibrinolysis; platelet activation; collagen biosynthetic process; protein amino acid phosphorylation; proteolysis; response to stress; wound healing
6	IPI00326984	Inter-alpha-trypsin inhibitor heavy chain H3	Itih3	**1.84±0.52**	**1.73±0.25**	**2.11±0.57**	**1.01±0.38**	**2.0**	**1.4**	hyaluronan metabolic process
7	IPI00568410	Protein kinase C-binding protein	Nell2	**0.87±0**	**0.26±0**	**0.59±0**	**0.59±0**	**0.7**	**0.4**	cell adhesion; regulation of growth
8	IPI00200257	T-cadherin	Cdh13	**0.57±0**	**0.28±0**	**0.77±0**	**0.56±0**	**0.7**	**0.4**	Rac protein signal transduction; Rho protein signal transduction; LDL-mediated signaling; EGFR signaling pathway; cell adhesion; cell growth and proliferation; calcium-mediated signaling; cell migration; chemotaxis; survival gene product expression; endocytosis; response to organic substance; sprouting; angiogenesis
9	IPI00209114	Isoform 1 of Limbic system-associated membrane protein	Lsamp	**0.77±0**	**0.46±0**	**0.53±0**	**0.45±0**	**0.7**	**0.5**	cell adhesion; nervous system development
10	IPI00199030	Neuroendocrine protein 7B2	Scg5	**0.51±0**	**0.52±0**	**0.47±0.07**	**0.36±0.03**	**0.5**	**0.4**	intracellular protein transport; neuropeptide signaling pathway; peptide hormon processing; regulation of hormone secretion; transport
11	IPI00391338	Similar to ICOS ligand precursor	Icosl	**1.12±0**	**0.39±0**	**0.59±0**	**0.63±0**	**0.9**	**0.5**	positive regulation of interleukin-4 biosynthetic process

Proteins consistently changed across two LC-MS/MS runs.

Potential biomarkers were selected based on average ratio ≥2.0 or ≤0.5. SD – standard deviation. M/S – moderate/sham ratio; S/S – severe/sham ratio.

**Table 3:** Potential biomarkers of SCI severity identified by quantitative LC-MS/MS analysis of CSF from rats following experimental SCI.

	Protein ID	Protein Description	Protein Symbol	Moderate/sham		Severe/sham		Gene Ontology
				AVG ratios	SEM	AVG ratios	SEM	
1	IPI00188541	Inter-alpha-inhibitor H4 heavy chain	Itih4	**4.5**	0.41	**1.8**	0.33	hyaluronan metabolic process; acute phase response
2	IPI00476458	Glutathione peroxidase 3	Gpx3	**4.2**	0.30	**1.9**	0.27	glutathione metabolic process; hydrogen peroxide catabolic process; oxidation reduction; protein homotetramerization; response to oxidative stress
3	IPI00326140	Pregnancy zone protein	Pzp	**3.9**	0.41	**2.1**	0.36	pregnancy
4	IPI00360930	Carbonic anhydrase 1	Car1	**2.4**	0.21	**0.4**	0.12	one-carbon metabolic process
5	IPI00207146	Zero beta-1 globin	MGC72973	**6.1**	0.78	**1.0**	0.31	oxygen transport
6	IPI00191715	Alpha-1-acid glycoprotein	Orm1	**2.1**	0.18	**0.9**	0.21	acute-phase response; regulation of immune system process

Proteins consistently changed across three LC-MS/MS runs.

Selection of potential biomarkers of SCI severity was based on more than two-fold difference in the average ratio between the two injury models. AVG – average; SEM – standard error of the mean.

**Table 4:** Potential biomarkers of SCI severity identified by quantitative LC-MS/MS analysis of CSF from rats following experimental SCI.

	Protein ID	Protein Description	Protein Symbol	Run 1 ratios (±SD)		Run 2 ratios (±SD)		Average ratios		Gene Ontology
				M/S	S/S	M/S	S/S	M/S	S/S	
1	IPI00231370	Protein S100-A8	S100a8	**5.32±0**	**11.8±0**	**0.95±0.68**	**3.31±2**	**3.1**	**7.6**	Chemotaxis; inflammatory response
2	IPI00201561	Peroxiredoxin-2	Prdx2	**3.08±0.13**	**0.36±0.11**	**2.45±1.61**	**0.44±0.19**	**2.8**	**0.4**	T cell proliferation and differentiation; activation of MAPK activity; anti-apoptosis; cell homeostasis; hydrogen peroxide metabolism; negative regulation of NFkB activity; lipopolysaccharide-mediated signaling pathway; neuron apoptosis; respiratory burst during acute inflammatory response; response to oxidative stress; thymus development
3	IPI00230787	Carbonic anhydrase 2	Car2	**2.53±0**	**0.76±0**	**2.60±0**	**0.41±0**	**2.6**	**0.6**	CO_2_ transport; kidney development; epithelium morphogenesis; odontogenesis; one-carbon metabolic process; osteoclast differentiation; bone resorption; regulation of cellular pH; response to steroid hormone stimulus; response to stress; response to zinc ion; secretion
4	IPI00558944	RGD1564861 similar to Ig variable region, light chain	RGD1564861	**1.28±0**	**3.32±0**	**2.00±0.1**	**4.86±0.11**	**1.6**	**4.1**	rat plasma protein; immune response

Proteins consistently changed across two LC-MS/MS runs.

Selection of potential biomarkers of SCI severity was based on more than two-fold difference in the average ratio between the two injury models. SD – standard deviation; M/S – moderate/sham ratio; S/S – severe/sham ratio.

**Figure 4:**
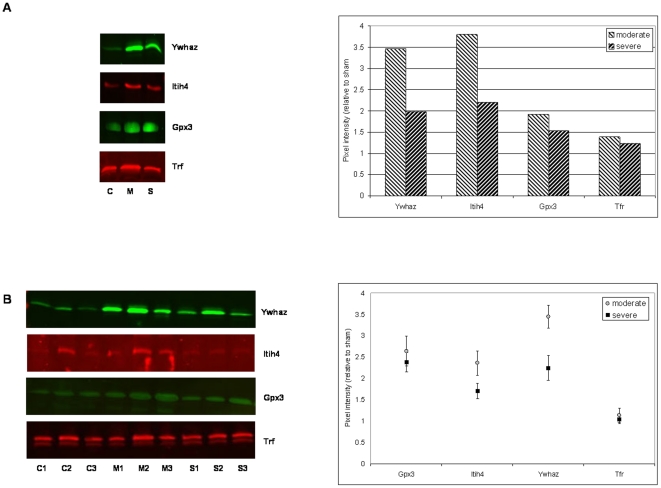
Western blot validation of candidate biomarkers of spinal cord injury. (a) pooled sample of CSF from 12 sham injury rats (C); pooled sample of CSF from 12 moderate injury rats (M); pooled sample of CSF from 12 severely injured rats (S). The chart on the right shows quantification of the Western blots of moderately and severely injured rats relative to sham injury, (b) three pools of CSF from four sham, moderate and severe injury rats, C1–C3, M1–M3, S1–S3, respectively. The chart on the right shows quantification of the Western blots relative to sham injury. Each data point represents mean of three samples; error bars – standard error of the mean.

Based on protein ratios, biological function, utility as a marker of severity, and antibody availability, we selected four proteins for validation by Western blot analysis: Ywhaz; Gpx3; Itih4; and S100a8. Serotransferrin (Tfr, IPI00679202), which was not affected by the injury in LC-MS/MS analysis, was included in the Western blot analysis as a control. For this analysis the animal injury experiments were repeated and CSF was collected. The limited amount of CSF obtained from a single rat prevented analysis of individual CSF samples. However, to obtain some information about the degree of variability within each injury group the CSF samples were pooled randomly to create three independent CSF samples for each injury group. The western blot results confirmed the MS data for all of the proteins, except for S100a8 (Figure 4). S100a8 was not detectable in CSF by WB even though it was detected in a spleen sample, which was run in parallel as a positive control. We observed some degree of variability in the proteins levels between the CSF samples within each injury group. However, the overall trend in protein change was conserved, with all three proteins showing increased levels after injury (Figure 4b). Furthermore, there was a significant difference in the Ywhaz and Itih4 protein levels between moderate and severe injury groups, confirming their potential to serve as markers of injury severity (Figure 4b quantification pane).

**Figure 5:**
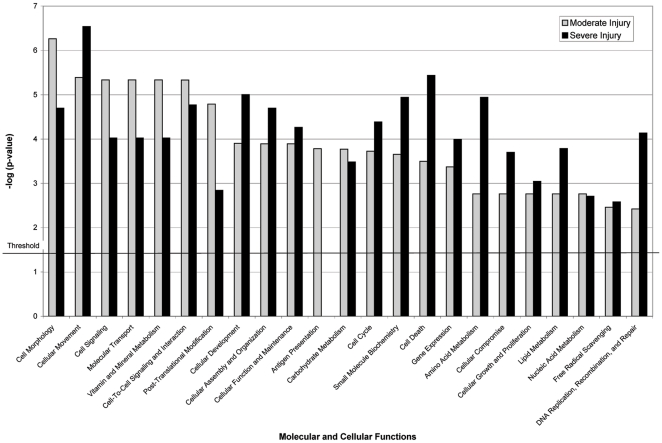
Results of LC-MS/MS data analysis using core module of the Ingenuity Pathways Analysis (IPA) software package. The LC-MS/MS data set was filtered for proteins present in CSF only. The proteins from this filtered dataset that met the 1.5 fold cut-off criteria and were associated with biological functions in the IPA Knowledge Base were considered for the analysis. A p-value determining the probability that each biological function assigned to that data set is due to random chance alone was calculated using right-tailed Fischer's exact test. P-values<0.05 were considered statistically significant.

To determine if molecular and cellular processes are differentially affected in the moderate vs. severe models of SCI, we analyzed our LC-MS/MS data set using the “Core analysis” module of the IPA software. The IPA program facilitates the interpretation of expression data in the context of biological systems by associating the set of differentially expressed genes with known (experimentally demonstrated) biological relationships. For this analysis we used the “CSF fluid” as the dataset filter and a 1.5-fold change in protein abundance as a cut-off value. In the context of a biological system, modest changes in the levels of a large number of proteins involved in a particular pathway or biological process may be more meaningful than large changes in a small number of proteins. This was the rationale for selecting less stringent cut off values for the IPA analyses (1.5-fold for the IPA vs. 2.0-fold for the identification of potential biomarkers of SCI). Using this selection criteria a total of 76 CSF proteins were consistently affected by SCI. Although comparison of the IPA functional analyses of moderate and severe injury data sets indicates that many molecular and cellular processes are significantly (p<0.05) affected in both injury models, proteins involved in cellular and molecular functions such as cell morphology, cell signaling, antigen presentation, molecular transport, and vitamin and mineral metabolism were associated more significantly with the moderate injury data set (Figure 5). Whereas, changes in the abundance of proteins associated with cellular movement, development, macromolecular assembly, cellular organization, cell death, small molecule biochemistry, and amino acid and lipid metabolism were more evident in the severe injury data set.

## Discussion

To find biomarkers of SCI severity we used a standardized rat injury model in which an electro-mechanic impactor strikes the spinal cord and displaces it to a specific extent. This allowed us to define the severity of injury (moderate vs. severe injury) and resulted in a reasonable consistency of injury within each cohort of test animals. Through LC-MS/MS analyses of labeled CSF peptides we identified a total of 42 proteins that are potential biomarkers of spinal cord injury. Importantly, 10 of these proteins allow one to distinguish between moderate and severe SCI events. Western blot analyses confirmed the MS results for Gpx3, a potential SCI biomarker, and two potential markers of SCI severity, the Itih4 and Ywhaz.

Several immune response proteins, including B2M, Serpinc1, A2M, C3, ApoA1, ApoH, and Ambp, are present among the potential SCI biomarkers identified in our study. The immune proteins are often thought to be plasma-derived proteins, and one might argue that their presence in the CSF following SCI may be due to leakage through the blood-CNS barrier. However, the fact that higher levels of these proteins are present in the moderate injury CSF, where there is less damage to the blood-CNS barrier, argues against plasma leakage as a source of these proteins in CSF. Moreover, the absence of ApoB, which is not normally present in CSF and represents a marker of plasma leakage [25], makes leakage from plasma an unlikely source for the increased abundance of immune proteins in our experiments.

The observation that the relative abundance of some of these proteins is higher among those animals with moderate injury than it is among those with severe injury seems somewhat counterintuitive. However, many of these proteins, including Apoa1 [26], Serpinc1 [27], Ambp [28], A2M [29] are negative regulators of the immune response. Since cells in damaged tissue must choose one of two options: either cellular repair or cellar elimination, we speculate that in moderate injury, many of the cells engage the cellular repair pathway, and suppression of some components of the immune response would be consistent with this process. If that were true, one might predict that proteins that stimulate the immune response would be up-regulated among those animals that experienced a severe SCI event. Indeed, two of the proteins that were up-regulated in the severely injured animals, S100a8 and B2M, are known to function as positive regulators of the immune response [30], [31]. Interestingly, Khwaja et al [32] recently reported a similar observation in patients with brain tumors. They noted that many immune response proteins were elevated in the CSF from patients with lower grade astrocytoma but were absent from CSF of patients with high-grade tumors.

The levels of two antioxidant enzymes, Gpx3 and Prdx2, were considerably higher in the CSF of animals that had suffered a moderate SCI as compared to those animals that had a severe SCI. Furthermore, Prdx2 levels were down-regulated in severe and up-regulated in moderate injury group as compared to sham. This suggests that the cellular anti-oxidant response mechanisms may be weaker among animals that were subjected to severe SCI. This finding is particularly interesting, since oxidative stress is a well documented mechanism of secondary injury following traumatic SCI [33], [34], [35]. If this is true, then the oxidative stress response mechanism may provide an interesting therapeutic target, since the reduction in the level of oxidative stress may effectively reduce some of the protein turnover within the cells. In turn, this may reduce the progression of the secondary damage that normally accompanies SCI, and thus enhance neurological recovery.

The identification of two 14-3-3 proteins, Ywhaz and Ywhag, among the potential SCI biomarkers came as no surprise. The 14-3-3 proteins are ubiquitously expressed, with their highest levels found in CNS tissues. These proteins are able to bind a multitude of different signaling proteins including kinases, phosphatases and transmembrane receptors and play a major role in modulating a wide range of regulatory processes such as mitogenic signal transduction, cell cycle control, ion channel regulation, and apoptosis [36]. Elevated levels of the 14-3-3 proteins in the CSF are associated with acute brain injury [37], dementia [38], Creutzfeldt-Jakob disease [39], and motor neuron injury [40]. Therefore, it is quite likely that their elevated levels following SCI reflect the process of neuronal damage [39]. With the exception of slightly higher levels of Ywhaz in the CSF after severe injury as compared to moderate injury (a finding confirmed by western blot), we did not observe a significant difference in protein levels of these two 14-3-3 proteins between the two injury models. Taken together, these two observations suggest that elevated levels of Ywhaz and Ywhag proteins may be useful as general markers of neuronal injury, including SCI, but their levels do not distinguish differing severities of injury.

It is worth noting the presence of three inter-alpha-trypsin inhibitors (Itih1, Itih3, and Itih4) in our panel of candidate biomarkers of SCI. In addition, ITIH4 is a potential biomarker for the severity of SCI. The ITIs are a family of structurally related plasma serine protease inhibitors that are involved in extracellular matrix stabilization and the inflammatory response [41]. They have been shown to bind hyaluronan (HA), a glycosaminoglycan (GAG), which is an integral component of the CNS extracellular matrix and thus modulate the levels of free HA [42]. Free HA accumulates in the extracellular matrix of damaged CNS [43], [44] and may contribute to the inhibition of re-myelination and axonal regeneration during CNS repair due to its influence on neural stem/progenitor cell differentiation, astrocyte proliferation and migration, oligodendrocyte maturation and re-myelination, axon outgrowth and the onset of inflammation [45]. Hence higher levels of inter-alpha-antitrypsin inhibitors in the moderate injury CSF may reduce the amount of free HA and limit its inhibitory effect on spinal cord repair. Accordingly, this may suggest a pathway, and thus potential targets, for the development of compounds that suppress this inhibitory process.

The S100ß and NSE proteins were previously reported as potential biomarkers of traumatic SCI in rat [8], [9]. However, we did not find any significant alterations in the relative abundance of these proteins in our experiments. Of course, it is possible that both of these proteins are elevated within the first few hours of SCI, with levels peaking at 6 h post injury and are not detectable after 24 hours in rats [8], [9]. This appears to be the case also in patients that receive thoracic endovascular stent grafting to treat thoracic and thoracoabdominal aortic aneurysms (TAAAs), a procedure associated with an increased risk of ischemic spinal cord damage [46], [47]. However, our LC-MS/MS analyses did identify another member of the S100 family of calcium binding proteins, S100a8, and its level appears to be correlated with the severity of SCI. Our attempt to confirm this result by WB was unsuccessful, possibly because the S100a8 levels were too low to be detected by WB analyses using this particular antibody. The S100a8 protein belongs to the S100 family of proteins involved in the regulation of calcium dependent intracellular processes and is implicated in diverse cellular and molecular processes including protein phosphorylation, transcription, cell-cycle regulation, cell growth, motility, differentiation and survival, as well as, inflammation [30] [47]. Hence, even though we were unable to validate this potential biomarker of SCI severity by WB, we feel that this protein needs to be investigated further, with more extensive sample pre-fractionation prior to quantification.

Applying the Ingenuity Pathways Analysis (IPA) to the LC-MS/MS data revealed a differential effect on several molecular and cellular processes in the two SCI injury models. Interestingly, changes in protein levels within the severe injury data set showed a stronger association with cellular processes such as cell movement, development, assembly, organization, and death. This association was primarily due to down-regulation of several proteins involved in these processes, suggesting a shutdown of cellular functions. Among these proteins Prdx2, Ncam1, and Chl1 have also been shown to decrease neuronal cell death [48], [49], [50]. Furthermore IPA analyses indicated that some metabolic processes are also differentially affected in the two injury models, with the moderate injury dataset showing stronger association with calcium transport and calcium efflux/influx, and the severe injury dataset being more significantly associated with lipid metabolism. Lipid signaling plays a crucial role in the development, differentiation, function, protection, and repair of the CNS and deregulation of lipid metabolism is believed to be a key event in neurodegenerative disorders and CNS injuries such as stroke [51]. Loss of intracellular calcium homeostasis is also a contributing factor in the development of neurodegenerative disorders and CNS injuries, including stroke and SCI, and an imbalance in calcium handling can intensify the susceptibility of neural tissue to oxidative stress and can lead to mitochondrial dysfunction resulting in cell death [51], [52].

LC-MS/MS analysis of CSF protein levels after SCI generated a panel of potential biomarkers of SCI, including a set of markers that may help to distinguish between moderate vs. severe SCI events. One limitation of this study was the small amount of CSF obtained from a single rat, which prevented the analysis of individual animals to determine the degree of inter-individual variability in the proteins levels following injury. Notwithstanding the need for sample pooling, our data provides support for the hypothesis that differences in protein levels may reflect the severity of injury to the spinal cord, and hence could be used as biomarkers to determine the severity of SCI and allow neurological prognosis.

Another limitation of the study is the presence of only one time point for the collection of CSF after spinal cord injury. Inclusion of additional time points would provide more information about the role of identified putative markers in the secondary spinal cord damage. Also, given the complex and dynamic pathology of SCI, it can be expected that the levels of biomarkers of spinal cord damage will be time dependent, and thus an accurate assessment of the severity of the injury will require a marker that is stably altered during the acute phase of SCI. More likely, a panel of several markers potentially at different times post-injury will provide a better correlation with injury severity. However, the main goal of this study was to determine the feasibility of employing mass spectrometry to detect biomarkers of SCI and further, whether this technique could reveal objective biomarkers of the severity of SCI, hence we only examined one time point. Since we detected 42 potential biomarkers, ten of which may distinguish injury severity, we believe the method has been confirmed. Initially, we chose the 24 hour post-injury collection of CSF because it is a time point at which it will be feasible to obtain CSF from injured patients – many of whom arrive at the hospital from hundreds of miles away. Future studies will undoubtedly canvas several post-injury time points which, in addition to providing biomarkers of SCI, will provide insight into the repair mechanisms, provide targets for therapeutic intervention and allow monitoring of patient recovery.

## Supporting Information

Table S1 Peptides identified in the cerebrospinal fluid of rat.
(XLS)

Table S2 Proteins identified in the cerebrospinal fluid of rat.
(XLS)

Table S3 CSF protein ratios after moderate or severe spinal cord injury. Protein ratios are relative to protein levels in CSF from sham animals. M/C - moderate/sham; S/C - severe/sham.
(XLS)
